# A Nine-Gene Signature for Predicting the Response to Preoperative Chemoradiotherapy in Patients with Locally Advanced Rectal Cancer

**DOI:** 10.3390/cancers12040800

**Published:** 2020-03-26

**Authors:** In Ja Park, Yun Suk Yu, Bilal Mustafa, Jin Young Park, Yong Bae Seo, Gun-Do Kim, Jinpyo Kim, Chang Min Kim, Hyun Deok Noh, Seung-Mo Hong, Yeon Wook Kim, Mi-Ju Kim, Adnan Ahmad Ansari, Luigi Buonaguro, Sung-Min Ahn, Chang-Sik Yu

**Affiliations:** 1 Department of Surgery, Asan Medical Center, University of Ulsan College of Medicine, Seoul 05505, Korea; ipark@amc.seoul.kr; 2 CbsBioscience Inc., Daejeon 34036, Korea; yuyunsuk@cbsbio.com (Y.S.Y.); jonnypark@cbsbio.com (J.Y.P.); ybseo@cbsbio.com (Y.B.S.); gundokim@cbsbio.com (G.-D.K.); kjp@cbsbio.com (J.K.); kcm3879@cbsbio.com (C.M.K.); nhd@cbsbio.com (H.D.N.); 3 Department of Health Sciences and Technology, Gachon Advanced Institute for Health Sciences and Technology, Gachon University, Incheon 21565, Korea; bmustafa12@gc.gachon.ac.kr; 4 Department of Microbiology, College of Natural Sciences, Pukyong National University, Busan 48513, Korea; 5 Department of Pathology, Asan Medical Center, University of Ulsan College of Medicine, Seoul 05505, Korea; smhong28@gmail.com; 6 Asan Institute for Life Science, Asan Medical Center, University of Ulsan College of Medicine, Seoul 05505, Korea; dusnrl486@naver.com (Y.W.K.); kimmiju@nate.com (M.-J.K.); 7 Department of Industrial and Environmental Engineering, Graduate School of Environment, Gachon University, Incheon 21565, Korea; adnanansa@gmail.com; 8 Cancer Immunoregulation Unit, Istituto Nazionale per lo Studio e la Cura dei Tumori, “Fondazione Pascale”-IRCCS, 80131 Naples, Italy; l.buonaguro@istitutotumori.na.it; 9 Department of Genome Medicine and Science, College of Medicine, Gachon University, Incheon 21565, Korea

**Keywords:** biomarker, locally advanced rectal cancer, preoperative chemoradiotherapy, NanoString analysis

## Abstract

Preoperative chemoradiotherapy (PCRT) and subsequent surgery is the standard multimodal treatment for locally advanced rectal cancer (LARC), albeit PCRT response varies among the individuals. This creates a dire necessity to identify a predictive model to forecast treatment response outcomes and identify patients who would benefit from PCRT. In this study, we performed a gene expression study using formalin-fixed paraffin-embedded (FFPE) tumor biopsy samples from 156 LARC patients (training cohort *n* = 60; validation cohort *n* = 96); we identified the nine-gene signature (*FGFR3*, *GNA11*, *H3F3A*, *IL12A*, *IL1R1*, *IL2RB*, *NKD1*, *SGK2*, and *SPRY2*) that distinctively differentiated responders from non-responders in the training cohort (accuracy = 86.9%, specificity = 84.8%, sensitivity = 81.5%) as well as in an independent validation cohort (accuracy = 81.0%, specificity = 79.4%, sensitivity = 82.3%). The signature was independent of all pathological and clinical features and was robust in predicting PCRT response. It is readily applicable to the clinical setting using FFPE samples and Food and Drug Administration (FDA) approved hardware and reagents. Predicting the response to PCRT may aid in tailored therapies for respective responders to PCRT and improve the oncologic outcomes for LARC patients.

## 1. Introduction

Locally advanced rectal cancer (LARC) is described as an invasive rectal tumor with unresectable margins involving the mesorectal fascia and clinically suspicious lymph nodes (lateral pelvic lymph nodes) [1,2]. Preoperative chemoradiotherapy (PCRT) followed by surgical resection is the standard multimodal treatment for LARC [3,4]. Around 30% of the total patients show a complete response to this treatment, having better long-term oncologic results and local control [5,6,7,8,9,10,11]. PCRT decreases the risk of local recurrence and increases the possibility of sphincter preservation. Patients who respond favorably to PCRT show good oncologic outcomes [12,13].

In patients that show a complete or near-complete response to PCRT, this treatment enables rectal-sparing surgical treatment of the tumor [14,15]. Although PCRT followed by surgical resection is the standard treatment for LARC, approximately two-thirds of patients show partial or no-response to PCRT; in these patients, PCRT does not improve the clinical outcome [16]. Moreover, PCRT is associated with two adverse effects in non-responders: (1) radiation therapy is associated with long-term complications that affect the quality of life of patients; and (2) delayed surgical resection due to PCRT may lead to local and distant tumor spread [17,18]. These outcomes have prompted extensive efforts to develop biomarkers for predicting the response to PCRT in LARC patients, which would enable the selection of responders who would benefit from PCRT [19].

Several studies demonstrated the potential of genetic biomarkers to accurately predict the response to and outcome of PCRT [6,20,21]. In 2005, Ghadimi et al. identified 54 differentially expressed genes (DEGs) between responders and non-responders, and expression profiling could predict tumor behavior in 83% of patients with LARC (*p* = 0.02) [22]. In 2014, Gantt et al. used 33 rectal cancer biopsy samples and identified two gene expression profiles that differentiated non-responders from responders [23]. Guo et al. recently identified a 27-gene signature in LARC patients capable of predicting the response to PCRT based on relative expression ordering (REO) patterns [24]. Chauvin et al. reported the potential of proteomic profiling for predicting the response to PCRT in LARC patients [25,26]. Despite improvements in our understanding and identification of gene expression profiles involved in LARC, biomarkers for clinical use have not been identified to date.

One of the factors limiting the development of PCRT biomarkers is the use of formalin-fixed paraffin-embedded (FFPE) biopsy samples collected before PCRT and surgical excision. However, fresh frozen samples cannot be used because of factors such as tumor cellularity and necrosis, and immune infiltrates limit downstream expression analyses.

Recent studies reported the results of gene expression analyses using FFPE samples at both the discovery and clinical stages. In 2019, Moratin et al. identified differentially expressed microRNAs (miRNAs) for risk stratification using oral squamous cell carcinoma FFPE tissues [27]. Pareira et al. used three groups of gastric FFPE samples to evaluate the expression profiles of 10 miRNAs as potential biomarkers of gastric cancer associated with field cancerization [28]. In 2018, Yamaguchi et al. identified a TP53 gene expression signature consisting of 33 genes by performing nCounter analysis on early breast cancer FFPE samples, demonstrating its prognostic power for predicting early-stage breast cancer [29].

The aim of the present study was to develop a clinically applicable gene signature for predicting the response to PCRT. The tumor regression grading (TRG) categories, which were selected according to the composition of the residual tumor and fibrosis, were as follows: (1) complete regression (no residual tumor cells and only a fibrotic mass), (2) near-complete regression (difficult to microscopically find residual tumor cells in the fibrotic tissue), (3) moderate regression (easily identifiable dominant irradiation-related changes with residual tumor), (4) minimal regression (a dominant tumor mass with obvious irradiation-related changes), and (5) no regression (no evidence of irradiation-related fibrosis, necrosis, or vascular changes). Study subjects were classified into two broad classes: responders (patients with complete or near-complete regression; *n* = 72) and non-responders (all other patients; *n* = 84). We used FFPE tissue samples for RNA extraction and performed gene expression analysis using Food and Drug Administration (FDA) approved hardware and kits.

## 2. Results

### 2.1. Clinicopathological Features

Table 1 summarizes the clinicopathological features of the study cohorts. There were no statistically significant differences between the training cohort and the validation cohort.

### 2.2. Differential Gene Expression Analyses between the Responder and the Non-Responder Groups

In the training cohort, differential gene expression analysis between responders (*n* = 27) and non-responders (*n* = 33) showed that 47 out of 730 genes were differentially expressed with statistical significance (*p* < 0.05). Of these 47 genes, 42 were selected after univariate logistic regression (*p* < 0.05) from which, 14 genes were highly expressed in responders and 28 genes were highly expressed in non-responders (Supplementary Table S1).

### 2.3. Gene Signature Selection and Logistic Regression Analyses

A nine-gene signature was determined by k-fold cross validation of the 42 DEGs to identify the optimal gene combination. The nine genes were *FGFR3*, *GNA11*, *H3F3A*, *IL12A*, *IL1R1*, *IL2RB*, *NKD1*, *SGK2* and *SPRY2*. The nine-gene signature consistently showed accuracy (86.9%) in differentiating between responders and non-responders (sensitivity = 81.5% and specificity = 84.8% in the training cohort) after two-fold cross validation among all candidate gene signatures (Supplementary Table S2).

The association of the selected gene signature with clinicopathological features was investigated. Univariate analysis showed that gender and the selected gene signature were significantly and positively correlated with PCRT response. Pathological T-stage was significantly negatively correlated with PCRT response (Table 2).

Multivariate analysis was performed to assess the association between the potential gene signature, gender and pathological T-staging. The results confirmed that the gene signature, gender and pathological T-staging were independent predictors of PCRT response (Table 3).

### 2.4. Selected Gene Signature Validation

The selected gene signature distinguished PCRT responders from non-responders with an accuracy of 81.0% in our validation cohort (*n* = 96) (Table 4).

The nine-gene signature predictive of PCRT response was highly related to KEGG signal transduction pathways including cancer-related pathways, *PI3K-Akt* signaling pathways (Supplementary Figures S1, S2), proteoglycans in cancer, human cytomegalovirus infection, and human papillomavirus infection. High interaction frequency genes related to the gene signature included *GRB2*, *HSP90AA1*, and *HSP90AB1* (Table 5).

## 3. Discussion

In this study, we identified and validated a nine-gene signature capable of predicting the response to PCRT in LARC patients. This nine-gene signature has three main advantages over previously reported predictive signatures: (1) it showed a better accuracy for predicting the response to PCRT than previously reported signatures [22,30,31]; Ghadimi et al. reported the gene expression profiling based on microarray, showing lower performances in sensitivity, specificity and accuracy which were 78%, 86% and 83% respectively, Zuo et al. described the gene combination based on RNA sequencing, showing lower performances in Area under the curve (AUC) of three-year and five-year survival rates, which were 0.711 and 0.683 respectively, Palma et al. also reported the genetic signature based on qRT-PCR, showing lower performance in sensitivity, specificity and accuracy which were 60%, 100% and 85% respectively; (2) gene expression analysis was performed using FFPE samples and FDA-approved hardware and reagents; and (3) the nine-gene signature was validated in larger cohorts than those used in previous studies [23,24,25]. In addition, the sensitivity and specificity of the test are two important factors in identifying the ability of a test to predict true positives (patients with disease) and true negatives (patients without disease) in a clinical setting. Our reported accuracy value determines a high proportion of true results with an accuracy of 86.9% and 81.0% in training and validation cohorts respectively. Therefore, this method is readily applicable to the clinical setting.

Several genes in our prognostic nine-gene signature have been identified as susceptible genes by various studies to have potential mechanistic roles in causing cancers, particularly in colorectal cancer (CRC). Several of them represent key members of intracellular signaling pathways and encode proteins that are responsible for cell proliferation, differentiation, apoptosis and angiogenesis [32,33]. An example of this is *FGFR3*, whose overexpression is prominently identified in CRC patients and identified to cause oncogenesis by uncontrollable cell proliferation and migration [34]. *GNA11* encodes the G protein’s alpha subunit and is involved in causing various cancers, particularly uveal melanoma [35,36]. However, it is reported that somatic and hotspot mutations lead to the constitutive activation of G proteins and can contribute to CRC [37]. *H3F3A* and *H3F3B* encode histone proteins and are also known for their mutations that cause cancers such as chondroblastoma and osteosarcoma [38]. A study reported the potential role of *H3F3B* gene in causing CRC due to over-activation of *MAPK* signaling pathway [39]. However, the role of the *H3F3A* gene in CRC still needs to be further investigated. Cytokines such as *IL12A*, *IL1R1* and *IL2RB*, which are identified in our gene signature, play a major role as inflammatory mediators and are reported to greatly contribute as one of the factors in CRC development [40]. A study reported a seven-gene expression profile based on 12 gene expression omnibus (GEO) datasets indicated that IL2RB is strongly associated with CRC [41]. Mutations in *NKD1* also lead to the activation of Wnt signaling pathways and the promotion of cell proliferation in CRC [33]. Similarly, *SPRY2* overexpression contributes to CRC through induction of epithelial-mesenchymal transition (EMT) cells [42]. *SGK1* has several roles in physiological processes such as migration and proliferation, and is reported to be upregulated in CRC [43]. *SGK2* is a paralog of *SGK1*; however, the mechanistic role of SGK1 in CRC still needs to be defined. To our knowledge, only a few of them have been identified as CRC prognostic biomarkers in previous studies. A major impediment could be the varying magnitude of experimental factors (e.g., sample size, different ethnicities, varying RNA-seq technologies). Therefore, our novel gene signature could greatly contribute to the panel of biomarkers in order to identify PCRT responders and non-responders in LARC patients, and is not only a practical tool for predicting the response to PCRT, but also a mechanistic link to the underlying biology of LARC.

The nine-gene signature for predicting the response to PCRT with high accuracy has two important clinical implications. First, good responders identified using the nine-gene signature can be treated with PCRT, which may lead to rectal-sparing surgery. Local excision or deferral of surgery is sometimes used to avoid surgical complications associated with radical resection and to reduce the risk of stoma formation, which may compromise quality of life. Second, the identification of poor responders would be beneficial for the treatment of patients with LARC because it would prevent exposure to toxic and inefficient radiation therapy in this population. In addition, the delay of surgical treatment because of ineffective PCRT could be avoided. This tailored approach based on the molecular characteristics of LARC could improve the overall survival and quality of life of patients with LARC.

In univariate and multivariate logistic regression, our nine-gene signature proved to be an independent feature for predictive value (Table 2 and Table 3). Our nine-gene signature showed better performance than the biomarkers based on microarray, RNA sequencing and qRT-PCR. The accuracy, specificity and sensitivity of the nine-gene signature could be improved by enlarging the cohort. 

## 4. Materials and Methods

### 4.1. RNA Extraction

Total RNA was extracted from FFPE tissues (*n* = 156) using the RNeasy FFPE kit (Qiagen, Hilden, Germany) with Deparaffinization Solution (Qiagen) and DNase I treatment (Qiagen). Written consent was obtained from patients before enrollment, and the study’s retrospective protocol was approved by the Institutional Review Board of Asan Medical Center (protocol number: 2017-0333). All authors had access to the study data and reviewed and approved the final manuscript.

### 4.2. Patients and Response Assessment

The study included 156 randomly selected rectal cancer patients (*n* = 156) divided into a training cohort (*n* = 60) and a validation cohort (*n* = 96). All patients underwent surgical resection after PCRT between January 2014 and December 2017 at Asan Medical Center, Seoul, South Korea. Patients were excluded if they did not undergo surgical treatment, had no available pretreatment biopsy specimens, or could not undergo post-treatment pathological response assessment.

Preoperative radiotherapy was delivered at a dose of 45–50.4 Gy in 25 or 28 fractions. Chemotherapy consisted of two cycles of intravenous 5-fluorouracil (375 mg/m2 daily) plus leucovorin (20 mg/m daily) in a bolus administered over 3 days during the 1st and 5th weeks of RT, or oral capecitabine (1650 mg/m daily) administered twice per day during radiotherapy. Surgical resection was performed 6–8 weeks after completion of PCRT and included local excision and radical resection performed according to the principles of total mesorectal excision [44].

The five-tier classification for tumor regression grading was used to evaluate the pathological responses of the primary tumor to PCRT [45]. The TRG categories, which were selected according to the composition of the residual tumor and fibrosis, were as follows: (1) complete regression (no residual tumor cells and only a fibrotic mass), (2) near-complete regression (difficult to microscopically find residual tumor cells in the fibrotic tissue), (3) moderate regression (easily identifiable dominant irradiation-related changes with residual tumor), (4) minimal regression (a dominant tumor mass with obvious irradiation-related changes), and (5) no regression (no evidence of irradiation-related fibrosis, necrosis, or vascular changes). Study subjects were classified into two broad classes: responders (patients with complete or near-complete regression; *n* = 72) and non-responders (all other patients; *n* = 84).

### 4.3. Gene Expression Assay

Gene expression analysis was performed using the nCounter PanCancer Pathway Panel (NanoString Technologies, Seattle, WA, USA). The panel analyzed 770 genes including 40 control genes, and each reaction contained 200 ng of total RNA in a 15 μL aliquot as well as reporter and capture probes. Quality control and normalization of the raw data were performed using nSolver Analysis Software v 3.0 (NanoString Technologies).

### 4.4. Statistical Analysis

The clinicopathological variables of the training and validation cohorts were evaluated using the χ2-test and Fisher’s exact test, and a *p*-value < 0.05 was considered statistically significant.

### 4.5. Statistical Combination Gene Analysis

All statistical analyses in this study were performed using the open source statistical programming environment R language (version 3.4.3). In the training cohort, the Student’s t-test was used to classify DEGs as over- or under-expressed (*p* < 0.05 and |fold-change| >1.5) to compare PCRT treatment responders with non-responders. DEGs were further shortlisted using univariate logistic regression (*p* < 0.05). The number of shortlisted DEGs analyzed in combination and the total number of gene combinations were calculated using the following formula:

$$\overset{n}{\underset{k=1}{\sum }}\frac{n!}{k!\left(n-k\right)!}$$

where *n* is the total number of shortlisted DEGs and *k* is the number of genes included in the combinations.

Multivariate logistic regression analysis was used to measure the association between gene signatures and clinicopathological features (*p* < 0.05; Table 3).

**Figure 1:**
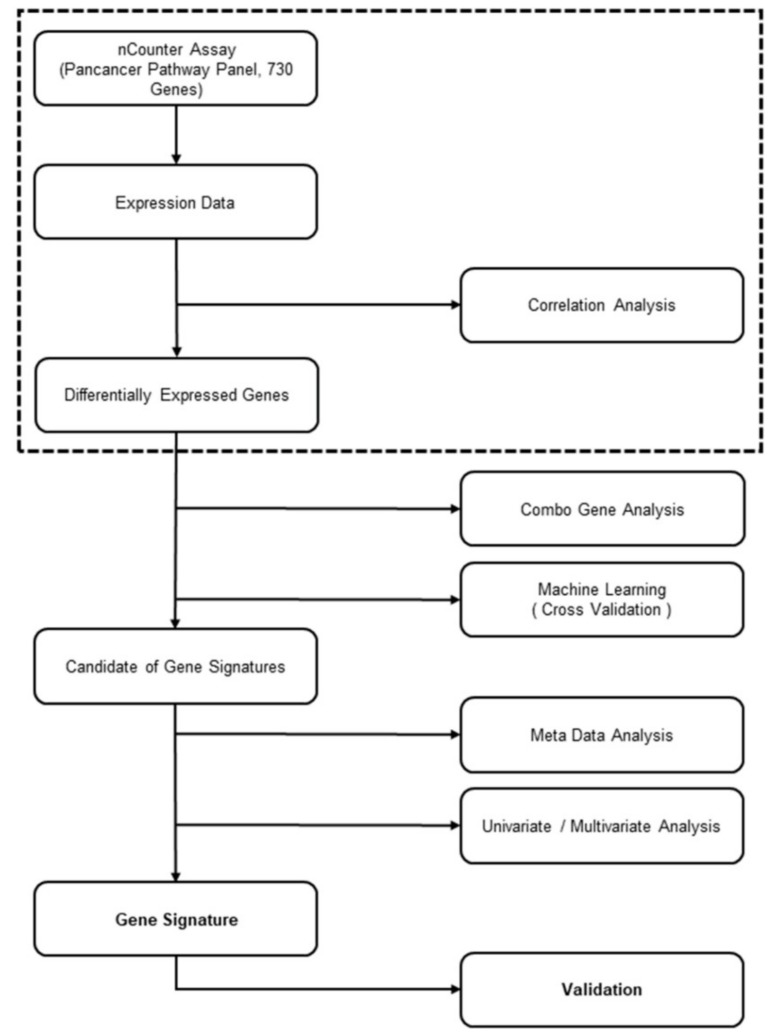
Gene signature development flowchart.

The candidate gene signatures (*p* < 0.05; AUC > 0.08; sensitivity > 75%; and specificity > 75%) were ranked by k-fold cross validation to identify the optimal gene combination. The training cohort was randomly separated by 2-fold (training set and test set) 300 times. The accuracy was calculated based on a *p*-value < 0.05 on the test set. The overall methodology is illustrated in Figure 1.

### 4.6. Comprehensive Causal Network Analysis Based on Meta-Analysis

A meta-analysis of the training cohort (*n* = 60) was performed to identify signal transduction pathways activated in PCRT responders. The meta-analysis was performed using CBS Probe PINGS™ (Protein Interaction Network Generation System; KR100957386B1; Daejon, Korea). The program uses five modules (protein–protein interactions, Path-finder, Path-linker, Path-maker, and Path-lister) to identify interacting genes and gene interaction information for gene combinations, such as interaction distance and interaction frequency. The identified genes were mapped to the signal transduction pathways obtained from the Kyoto Encyclopedia of Genes and Genomes (KEGG) database [46]. The top ten signal transduction pathways were selected according to the number of interactions and interacting genes.

A meta-analysis of the training cohort was performed to identify the most significant transduction pathways in PCRT responders by calculating the number of combination genes related to these signal transduction pathways.

**Figure 2:**
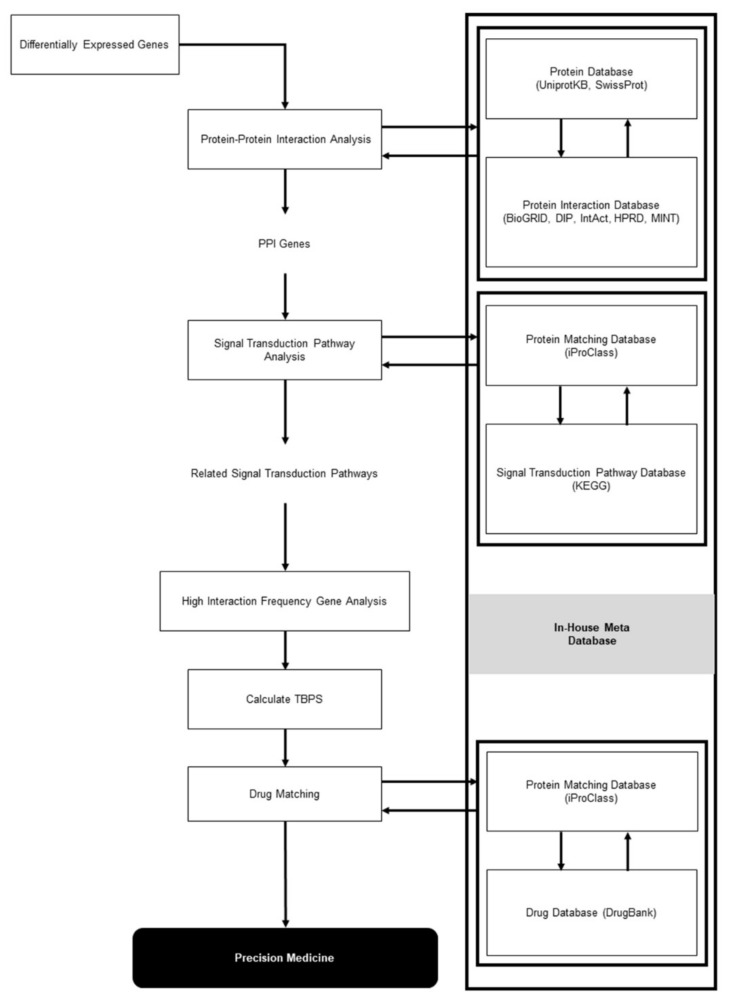
Meta-data analysis flowchart.

For each signal transduction pathway, we computed the gene interaction frequency of interacting genes with signature genes. A gene interaction frequency of 100% indicated the highest probability of gene interaction within each signal transduction pathway. A threshold of 75% was selected as a high interaction frequency (Figure 2). The top ten signal transduction pathways having high interaction frequency genes were identified in each patient in the training cohort, and gene signatures were matched to the related signal transduction pathways.

### 4.7. Data Availability

The NCBI GEO accession number for the data reported in this study is GSE139255.

## 5. Conclusions

In summary, we developed a nine-gene signature using DEG analysis, followed by k-fold cross validation and logistic regression models that robustly predicted the response to PCRT in patients with LARC. In addition, we applied meta-analysis to identify the most significant transduction pathways activated in PCRT responders. The prognostic value of the gene signature was statistically significant in all the datasets, while our meta-analysis results show that it is highly involved in *PI3K-Akt* pathways. Furthermore, this gene signature is readily applicable to the clinical setting using FFPE samples and FDA-approved hardware and reagents. This study suggests personalized treatment approaches in good and poor responders to PCRT, which ultimately improve the oncologic outcomes of patients with LARC.

## Figures and Tables

**Table 1:** Clinicopathological features of patients in the training and validation cohorts.

Variable	Training Cohort (*n* = 60)	Validation Cohort (*n* = 96)	*p*-Value
**Gender** Male Female	27 (45.0%) 33 (55.0%)	53 (55.2%) 43 (44.8%)	0.282
**Differentiation Grade** Well Moderately Poorly	9 (15%) 51 (85.0%) 0 (0.0%)	18 (18.9%) 76 (79.1%) 2 (2.0%)	0.596
**Clinical T-stage** T1 T2 T3 T4	0 (0.0%) 4 (6.7%) 53 (88.3%) 3 (5.0%)	0 (0.0%) 9 (9.4%) 78 (81.3%) 4 (9.0%)	0.810
**Clinical N-stage** N0 N1 N2	2 (3.3%) 24 (40.0%) 34 (56.7%)	10 (10.4%) 40 (41.7%) 46 (47.9%)	0.225
**Clinical M-stage** M0 M1	58 (96.7%) 2 (3.3%)	94 (97.9%) 2 (2.1%)	0.639
**Pathological T-stage** Tis T0 T1 T2 T3 T4	1 (1.7%) 9 (15.0%) 3 (5.0%) 17 (28.3%) 30 (50.0%) 0 (0.0%)	2(2.0%) 16 (16.7%) 4 (4.2%) 25 (26.0%) 46 (47.9%) 3 (3.1%)	0.908
**Pathological N-stage** N0 N1 N2	41 (68.3%) 10 (16.7%) 5 (8.3%)	66 (68.8%) 22 (22.9%) 8 (8.3%)	0.762
**Pathological M-stage** M0 M1	59 (98.3%) 1 (1.7%)	95 (99.0%) 1 (1.0%)	1.000

Abbreviations: T, tumor; N, node; M, metastasis.

**Table 2:** Univariate logistic regression analysis of the predictive value of the selected gene signature in preoperative chemoradiotherapy (PCRT) responders (*p* < 0.05).

Variable ^1^	N ^2^	Coef ^3^	SE ^4^ (Coef)	Z-Score	*p*-Value
**Candidate genes**					
*FGFR3_GNA11_H3F3A_IL12A_IL1R1_IL2RB_NKD1_SGK2_SPRY2* (low vs. high)	60	3.204371	0.693663	4.619491	3.85 × 10^−6^
**Clinicopathological features**					
GENDER (Male vs. Female)	60	1.170379	0.549265	2.130811	3.31 × 10^−2^
GRADED_DESCRIPTION (Moderate vs. Well)	59	−0.787079	0.783116	−1.00506	3.15 × 10^−1^
CLIN_T_TNM (T2 vs. T3-T4)	60	−1.386294	1.185853	−1.16903	2.42 × 10^−1^
CLIN_N_TNM (N0-N1 vs. N2)	60	−0.633724	0.528492	−1.19912	2.30 × 10^−1^
CLIN_M_TNM (M0 vs. M1)	60	16.8437	1696.73436	0.009927	9.92 × 10^−1^
PATH_T_TNM (Tis-T0-T1-T2 vs. T3)	60	−2.233592	0.605857	−3.68666	2.27 × 10^−4^
PATH_N_TNM (N0-N1 vs. N2)	56	−0.04879	0.956183	−0.05103	9.59 × 10^−1^
PATH_M_TNM (M0 vs. M1)	60	−15.39617	1455.39756	−0.01058	9.92 × 10^−1^

^1^ Abbreviations: CLIN, clinical; PATH, pathological; T, tumor; N, node; M, metastasis. ^2^ N, number of samples. ^3^ Coef, coefficient. ^4^ SE, standard error.

**Table 3:** Multivariate analysis of the association between the potential gene signatures and gender and pathological tumor staging (*p* < 0.05).

Variable 1	Odds Ratio	95% CI ^2^	*p*-Value
*FGFR3_GNA11_H3F3A_IL12A_IL1R1_IL2RB_NKD1_SGK2_SPRY2* (Low vs. High)	25.6	4.71–139.23	0.0002
GENDER (Male vs. Female)	2.26	0.48–10.72	0.3048
PATH_T_TNM (Tis-T0-T1-T2 vs. T3)	0.08	0.02–0.46	0.0042

^1^ Abbreviations: PATH, pathological; T, tumor; N, node; M, metastasis. ^2^ CI, confidence interval.

**Table 4:** Evaluation of the clinical performance of the nine-gene signature to predict PCRT response in patients.

Gene Signature		*FGFR3_GNA11_H3F3A_IL12A_IL1R1_IL2RB_NKD1_SGK2_SPRY2*
**Logistic regression *p*-value**		4.62 × 10^−4^
**Cross validation accuracy (%)**		83.3
**Number of genes**		9
**Training set**	Accuracy (%)	86.9
	Sensitivity (%)	81.5
	Specificity (%)	84.8
	Response (%)	81.5
**Validation set**	Accuracy (%)	81.0
	Sensitivity (%)	82.3
	Specificity (%)	79.4
	PPV ^1^ (%)	87.9
	NPV ^2^ (%)	71.1

^1^ PPV, positive predictive value. ^2^ NPV, negative predictive value.

**Table 5:** Gene signature-related pathways and high interaction frequency genes associated with PCRT responders.

Variable	Name of Pathways/High Interaction Frequency Genes
**Pathways**	Pathways in cancer *PI3K-Akt* signaling pathway Proteoglycans in cancer Human cytomegalovirus infection Human papillomavirus infection
**High interaction frequency genes**	*GRB2* *HSP90AA1* *HSP90AB1*

## Supplementary Materials

The following are available online at https://www.mdpi.com/2072-6694/12/4/800/s1, Figure S1: Metadata analysis-based PCRT responder gene signature related pathway: PI3K-Akt signaling pathway, Figure S2: PI3K-Akt signaling pathway three-dimensional genomic expression map for PCRT responders based on metadata analysis, Table S1: Differentially expressed genes between responders and non-responders in the training cohort, Table S2: Gene signature candidates.
