# Supplementary material for: A Nine-Gene Signature for Predicting the Response to Preoperative Chemoradiotherapy in Patients with Locally Advanced Rectal Cancer
Source: Cancers (Basel). 2020 Mar 26;12(4):800. doi: 10.3390/cancers12040800 (PMC7226472; doi:10.3390/cancers12040800)
Supplement: Supplementary file 1 [file cancers-12-00800-s001.pdf]

Supplementary Materials

NEDD3 belongs to the homologous to E6AP C-terminus (HECT) E3 ubiquitin ligase family. NEDD3 is overexpressed in CRC patients, and NEDD4 promotes colon cancer cell growth [1, 2].

The expression levels of the signal transducer and activator of transcription 1 and 3 (STAT1 and STAT3) were investigated in CRC biopsy samples of 400 patients [3]. The results suggested that STAT1 and STAT3 are effective prognostic biomarkers in CRC. In addition, activation of STAT3 promotes rectal cancer growth by inducing loss of PTPN4 [4].

TRAF6 is a member of the tumor-necrosis factor family and is involved in the NF-kappa B signaling pathway. Wu et al. showed that TRAF6 can effectively suppress CRC metastasis by promoting CTNNB1 degradation, suggesting its potential as a target for the treatment of advanced CRC [5].

HSP90AA1 and HSP90AB1 belong to the heat shock protein 90 family. Drug resistance caused by HSP90AA1-mediated autophagy was observed in chemotherapy-treated osteosarcoma [6]. Wang et al. suggested that HSP90AB1 could serve as a novel biomarker of poor prognosis in lung adenocarcinoma. The association of these genes with CRC remains to be established [7].

Table S1. Differentially expressed genes between responders and non-responders in the training cohort.

Sr. No.	Gene	*p*-value	Fold-Change *
1	ID1	2.17×10^{-2}	−2.78
2	WNT11	1.99×10^{-2}	−2.32
3	NKD1	1.90×10^{-3}	−2.27
4	SFN	2.45×10^{-3}	−2.24
5	LAMA5	8.51×10^{-3}	−1.96
6	CACNA1D	5.06×10^{-4}	−1.96
7	IKBKB	9.91×10^{-3}	−1.88
8	PLA2G4F	5.89×10^{-3}	−1.83
9	CDK6	3.63×10^{-3}	−1.79
10	CIC	1.21×10^{-2}	−1.76
11	SMARCB1	5.80×10^{-4}	−1.75
12	SRSF2	3.07×10^{-3}	−1.73
13	IKBKG	1.51×10^{-2}	−1.68
14	BAP1	9.01×10^{-3}	−1.68
15	SPRY2	1.52×10^{-3}	−1.65
16	AXIN2	5.01×10^{-3}	−1.65
17	GNA11	6.73×10^{-3}	−1.64
18	FGFR4	1.69×10^{-3}	−1.64
19	FGFR3	1.05×10^{-3}	−1.62
20	HDAC10	1.08×10^{-3}	−1.62
21	SGK2	5.64×10^{-3}	−1.6
22	H3F3A	2.42×10^{-3}	−1.58
23	EPHA2	1.74×10^{-2}	−1.57
24	U2AF1	4.56×10^{-3}	−1.56
25	ITGB4	3.14×10^{-3}	−1.56
26	TRAF7	6.07×10^{-3}	−1.54
27	CBL	5.56×10^{-3}	−1.53
28	MAP2K2	1.99×10^{-3}	−1.51

Sr. No.	Gene	*p*-value	Fold-Change *
29	BMP2	3.06×10^{-2}	1.5
30	CDC14B	5.16×10^{-3}	1.51
31	CACNA2D1	1.13×10^{-2}	1.52
32	ETV7	1.37×10^{-2}	1.55
33	STAT1	1.89×10^{-2}	1.56
34	ITGB3	2.98×10^{-2}	1.57
35	FAS	2.59×10^{-2}	1.58
36	IL1R1	2.31×10^{-2}	1.61
37	RASGRP1	3.42×10^{-2}	1.69
38	IRS1	1.17×10^{-2}	1.7
39	BMP8A	1.77×10^{-2}	1.75
40	IL12A	3.73×10^{-2}	1.99
41	CD40	1.51×10^{-2}	2.18
42	IL2RB	1.27×10^{-2}	2.33

* Fold-change was calculated by dividing the mean expression level of responders by that of non-responders.

Table S2: Gene signature candidates.

Sr. No.	Gene Signature	Logistic Regression p-value	Cross Validation Accuracy (%)	AUC [1]	Sensitivity (%)	Specificity (%)
**1**	**ETV7_H3F3A_HDAC10_ID1_IL1R1_SPRY2**	7.60×10^{-4}	72	0.841	77.8	81.8
**2**	**H3F3A_IL12A_IL1R1_NKD1_PLA2G4F_SGK2**	1.13×10^{-4}	79.7	0.868	77.8	87.9
**3**	**H3F3A_HDAC10_ID1_IL1R1_NKD1_SPRY2_STAT1**	1.81×10^{-4}	75.7	0.878	77.8	93.9
**4**	**H3F3A_HDAC10_IL1R1_PLA2G4F_SGK2_TRAF7_WNT11**	7.47×10^{-4}	57	0.822	81.5	75.8
**5**	**H3F3A_IL12A_IL1R1_NKD1_PLA2G4F_RASGRP1_SGK2**	1.99×10^{-4}	77.3	0.871	81.5	90.9
**6**	**FGFR3_H3F3A_HDAC10_ID1_IL1R1_IL2RB_TRAF7_WNT11**	4.65×10^{-4}	81	0.851	77.8	84.8
**7**	**FGFR3_GNA11_H3F3A_IL12A_IL1R1_IL2RB_NKD1_SGK2_SPRY2**	2.56×10^{-4}	83.3	0.869	81.5	84.8
**8**	**FGFR3_H3F3A_IL12A_IL1R1_IL2RB_NKD1_SGK2_SPRY2_TRAF7**	3.56×10^{-4}	73.7	0.870	77.8	84.8

[1] AUC; Area under the curve.

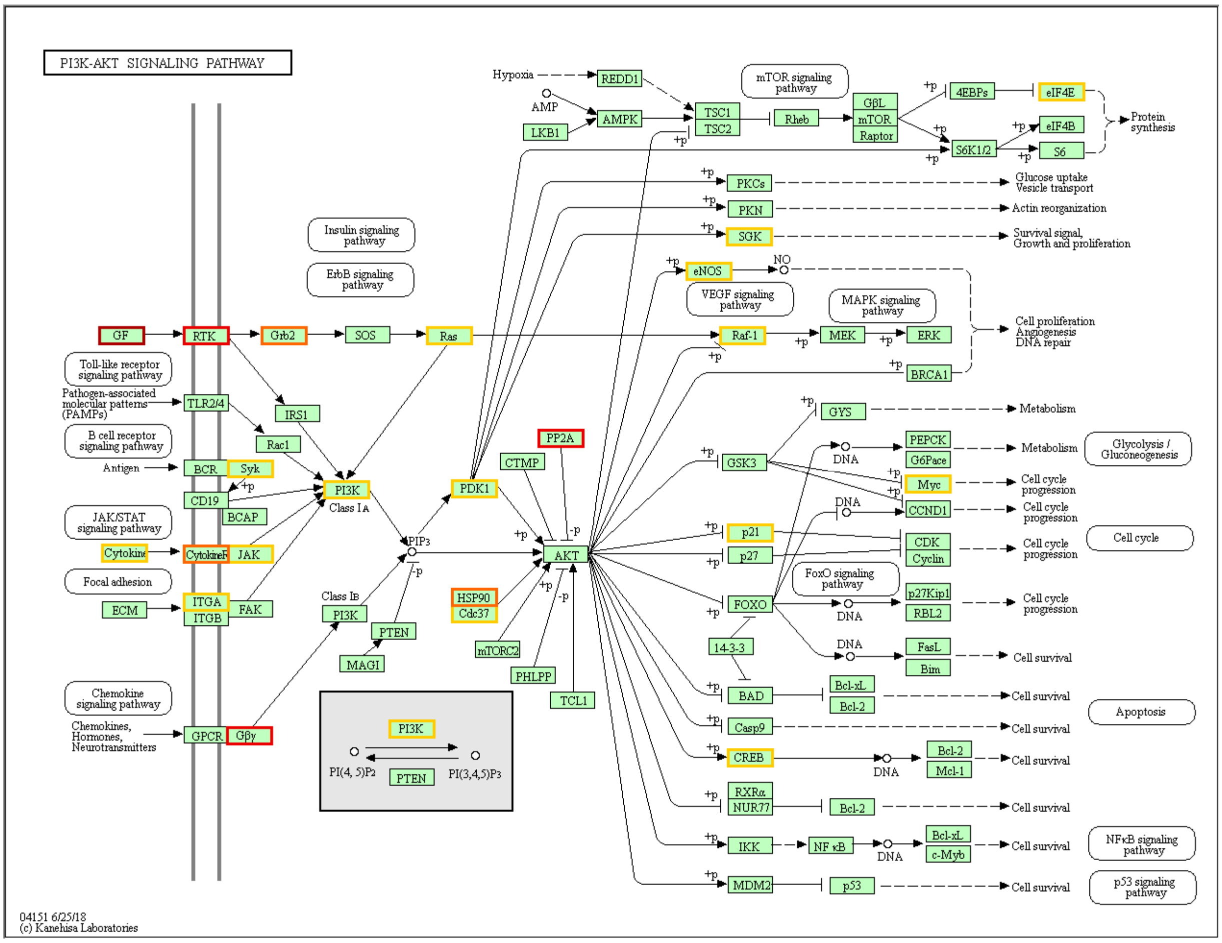

Figure S1. Metadata analysis-based PCRT responder gene signature related pathway: PI3K-Akt signaling pathway. In two-dimensional KEGG pathway maps, genes were assigned a color code according to the interaction frequency.

1

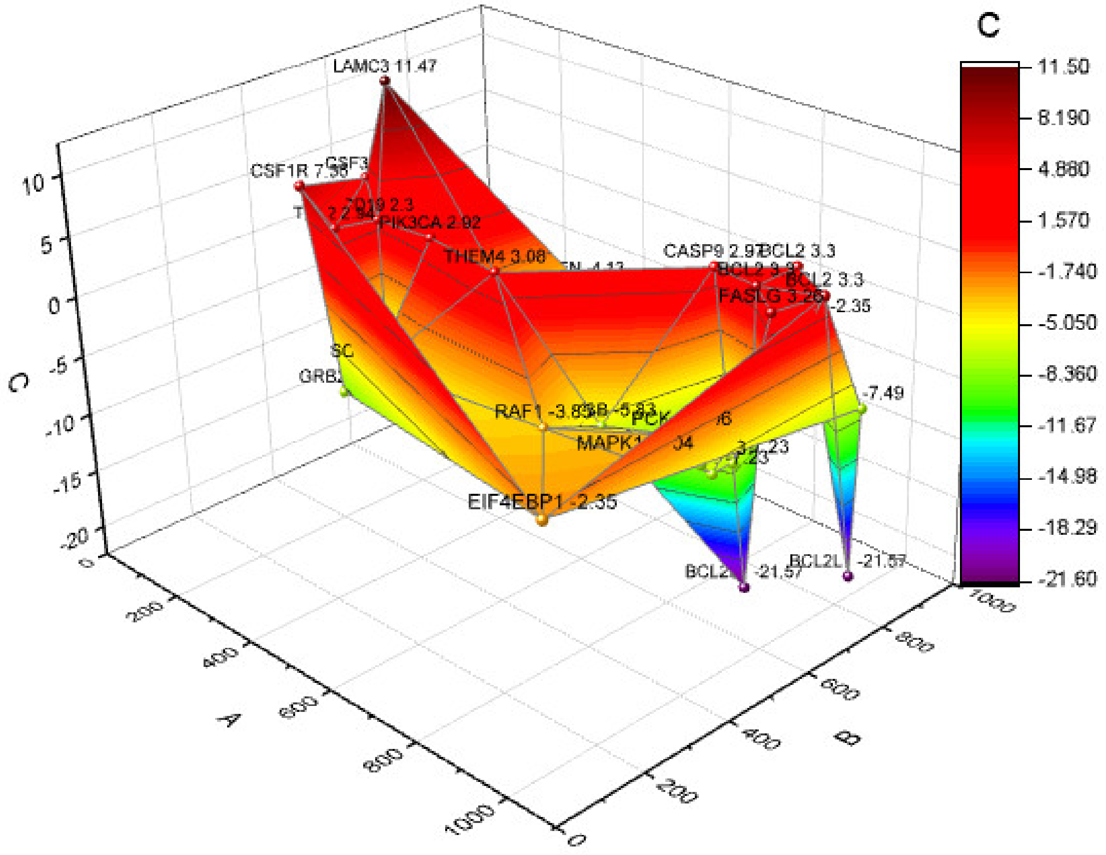

Figure S2. PI3K-Akt signaling pathway three-dimensional genomic expression map for PCRT responders based on metadata analysis. The gene regulatory networks associated with the response to PCRT in LARC patients were investigated by performing quantitative analysis of three-dimensional gene expression (gene signature-related high interaction genes). PI3K-Akt signaling pathway was identified as highly associated with gene signature related pathway in PCRT responders

References

1. Eide, P.W.; Cekaite, L.; Danielsen, S.A.; Eilertsen, I.A.; Kjenseth, A.; Fykerud, T.A.; Agesen, T.H.; Bruun, J.; Rivedal, E.; Lothe, R.A., et al. NEDD4 is overexpressed in colorectal cancer and promotes colonic cell growth independently of the PI3K/PTEN/AKT pathway. *Cell. Signal.* **2013**, *25*, 12–18, doi:10.1016/j.cellsig.2012.08.012.
2. Huang, Z.J.; Zhu, J.J.; Yang, X.Y.; Biskup, E. NEDD4 promotes cell growth and migration via PTEN/PI3K/AKT signaling in hepatocellular carcinoma. *Onc. Lett.* **2017**, *14*, 2649–2656, doi:10.3892/ol.2017.6532.
3. Gordziel, C.; Bratsch, J.; Moriggl, R.; Knosel, T.; Friedrich, K. Both STAT1 and STAT3 are favourable prognostic determinants in colorectal carcinoma. *Br. J. Cancer* **2013**, *109*, 138–146, doi:10.1038/bjc.2013.274.
4. Zhang, B.D.; Li, Y.R.; Ding, L.D.; Wang, Y.Y.; Liu, H.Y.; Jia, B.Q. Loss of PTPN4 activates STAT3 to promote the tumor growth in rectal cancer. *Cancer Sci.* **2019**, 10.1111/cas.14031, doi:10.1111/cas.14031.
5. Wu, H.; Lu, X.X.; Wang, J.R.; Yang, T.Y.; Li, X.M.; He, X.S.; Li, Y.; Ye, W.L.; Wu, Y.; Gan, W.J., et al. TRAF6 inhibits colorectal cancer metastasis through regulating selective autophagic CTNNB1/beta-catenin degradation and is targeted for GSK3B/GSK3beta-mediated phosphorylation and degradation. *Autophagy* **2019**, 10.1080/15548627.2019.1586250, doi:10.1080/15548627.2019.1586250.

6. Xiao, X.; Wang, W.; Li, Y.; Yang, D.; Li, X.; Shen, C.; Liu, Y.; Ke, X.; Guo, S.; Guo, Z. HSP90AA1-mediated autophagy promotes drug resistance in osteosarcoma. *J. Exp. Clin. Cancer Res.* **2018**, *37*, 201, doi:10.1186/s13046-018-0880-6.
7. Wang, M.; Feng, L.; Li, P.; Han, N.; Gao, Y.; Xiao, T. [Hsp90AB1 Protein is Overexpressed in Non-small Cell Lung Cancer Tissues and Associated with Poor Prognosis in Lung Adenocarcinoma Patients]. *Zhongguo fei ai za zhi* **2016**, *19*, 64–69, doi:10.3779/j.issn.1009-3419.2016.02.02.
